# Desire thinking as a predictor of compulsive sexual behaviour in adolescents: Evidence from a cross-cultural validation of the Hebrew version of the Desire Thinking Questionnaire

**DOI:** 10.1556/2006.2020.00062

**Published:** 2020-10-01

**Authors:** Yaniv Efrati, Daniel C. Kolubinski, Gabriele Caselli, Marcantonio M. Spada

**Affiliations:** 1 Faculty of Education and Society and Culture, Beit-Berl College, Kefar Sava, Israel; 2 Division of Psychology, School of Applied Sciences, London South Bank University, London, UK; 3 Sigmund Freud University, Milan, Italy

**Keywords:** adolescents, compulsive sexual behaviour, desire thinking, impulsivity, negative affect, thought suppression

## Abstract

**Background:**

Desire thinking is a voluntary cognitive process that involves the perseverative focus on memories, images and information related to a desired target. The aim of the present study was to validate the Hebrew version of the Desire Thinking Questionnaire (DTQ; Caselli & Spada, 2011) in a sample of adolescents and to investigate its relationship with measures of thought suppression, impulsivity and individual-based compulsive sexual behaviour.

**Methods:**

In Study 1, a convenience sample of 718 adolescents completed the newly translated Desire Thinking Questionnaire in Hebrew (DTQ-H) and results were subjected to an Exploratory Factor Analysis (EFA). In Study 2, a convenience sample of 379 adolescents completed a battery of questionnaires including the DTQ-H. A Confirmatory Factors Analysis was performed on the DTQ-H and validity was ascertained by correlating with other measures.

**Results:**

In Study 1, a 9-item two-factor structure was identified. A 6-item two-factor structure was confirmed in Study 2. Results also indicated that the DTQ-H has acceptable levels of reliability, and good concurrent and incremental validity in predicting compulsive sexual behaviour.

**Conclusions:**

The 6-item DTQ-H appears to be a reliable and valid measure of desire thinking and may be used also on adolescents – an understudied population.

## Introduction

### Desire thinking

Desire thinking is a voluntary cognitive process that involves the perseverative focus on memories, images and information related to a desired target (Caselli & Spada, 2011). This focus exists on a verbal and imaginal level, whereby the individual engages in repetitive self-talk regarding the need to achieve the desired target (termed verbal perseveration) and constructs mental images of the desired target or in its context of consumption (termed imaginal prefiguration; Caselli & Spada, 2011). The target of desire thinking may be an activity, such as drinking alcohol, gambling and smoking, the acquisition of an object, or the experience of a physiological or emotional state such as pornography craving (Kavanagh, Andrade, & May, 2005; Kavanagh, May, & Andrade, 2009; Salkovskis & Reynolds, 1994). A recent review and meta-analysis (Mansueto et al., 2019) has indicated that desire thinking was not examined, to date, in adolescents, although research has revealed that metacognition-related capacities improve with age during adolescence and reach a plateau only during adulthood (e.g. Weil et al., 2013). Therefore, the three aims of the current research were to: (1) validate one of the most extensively used questionnaires for desire thinking – the Desire Thinking Questionnaire (DTQ; Caselli & Spada, 2011) – in adolescents in order to open a new avenue for research on desire thinking; (2) determine the predictive capacity of the DTQ for compulsive sexual behaviour (CSB) independently of the established related cognitive and behavioural dispositions – thought suppression and impulsivity; and (3) explore the association between desire thinking and CSB that was found among adults in adolescents.

The objective of engaging in desire thinking is to shift attention away from craving in an attempt to lessen the discomfort produced by this experience (Caselli & Spada, 2010, 2013). However, engaging in desire thinking has the paradoxical effect of increasing the experience of craving, since the desired target is perseveratively fixated upon, but not achieved. This, in turn, leads to the desired target being perceived as the only route to attain relief from escalating distress (Caselli & Spada, 2011, 2015).

Research undertaken across numerous studies has shown that desire thinking is a transdiagnostic process as it is present across a range of targets, including alcohol (Caselli, Ferla, Mezzaluna, Rovetto, & Spada, 2012; Caselli, Gemelli, & Spada, 2017; Caselli & Spada, 2010), nicotine (Caselli, Nikčević, Fiore, Mezzaluna, & Spada, 2012; Caselli & Spada, 2010), food (Spada, Caselli, Fernie et al., 2015), gambling (Caselli & Spada, 2010; Fernie et al., 2014), problematic Internet (Spada, Caselli, Slaifer, Nikčević, & Sassaroli, 2014) social media use (Marino et al., 2019), and pornography (Allen, Kannis-Dymand, & Katsikitis, 2017; for a recent systematic review and meta-analysis see; Mansueto et al., 2019).

To date, the gold standard for assessing desire thinking is the 10-item DTQ (Caselli & Spada, 2011). The original version consists of two factors mentioned above, verbal perseveration and imaginal prefiguration. The DTQ has since been translated twice to date. Both the French version (Chakroun-Baggioni, Corman, Spada, & Caselli, 2017) and the Dutch version (Markus et al., 2019) confirmed the two-factor model, demonstrating adequate fit alongside the original.

Though many studies have investigated the role of desire thinking in addictive behaviours, employing cross-sectional, longitudinal and experimental designs (for a review see Mansueto et al., 2019), limited efforts have been directed at exploring the role of desire thinking in CSB. In a recent study desire thinking was linked with pornography craving among problematic pornography users (Allen et al., 2017). Specifically, it was found that both the imaginal prefiguration and verbal perseveration components of desire thinking were strongly linked to pornography craving. Imaginal prefiguration is characterised by the allocation of attentional resources to behaviour-related information, followed by mental imagery elaboration (e.g., imagination or memory recall) in anticipation of behavioural engagement; Verbal perseveration refers to extended self-talk regarding meaningful reasons for engaging in behaviour-related activities. Research has indicated that the higher the imaginal prefiguration (*r* = 0.44) and verbal perseveration (*r* = 0.51), the stronger the pornography craving. Of note, using mediation analysis, it was found that the effect of imaginal prefiguration on pornography craving was mediated by verbal perseveration, such that imaginal prefiguration was linked with higher verbal perseveration, which in turn was linked with pornography craving (i.e. imaginal prefiguration is only indirectly linked with pornography craving, whereas verbal perseveration is directly associated with this construct). Some researchers (e.g. Efrati, 2020) have argued that the problematic pornography use may be related to a broader disorder known as Compulsive Sexual Behaviour Disorder (CSBD) which is characterised by an extensive pornography use and masturbation, use of paid sexual services, and risky sexual behaviours and/or intense preoccupation with sex. These behaviours often lead to impaired social or occupational functioning, distress and negative affect (Kafka, 2010; Kraus et al., 2018). The prevalence of CSBD ranges between 3 and 10% among adults (Carnes, Green, & Carnes, 2010; Coleman, 1992; Dickenson, Gleason, Coleman, & Miner, 2018; Reid, 2013; SASH, 2008) and it is especially pronounced among adolescents such that between 12 and 18% are thought to have CSBD (Efrati & Dannon, 2018). Of note, these rates are not based on a nationally representative sample of adolescents and so may be taken with caution until nation-wide studies will replicate these figures. To date, however, desire thinking has not been investigated in relation to CSB and among adolescents. A recent systematic review and meta-analysis on the link between desire thinking and addictive behaviour (Mansueto et al., 2019) has indicated that most studies were conducted on adults (40 years and above) with only two studies on young adults (25 years and above) and no studies to date on adolescents. A recent study on the topic (Thomas, Katsikitis, Allen, & Kannis-Dymand, 2020) also solely examined adults. Studies have indicated that metacognition (which is linked with desire thinking) improves significantly with age during adolescence, is highest in late adolescence (ages 18–20) and reaches a plateau going into adulthood (e.g. Weil et al., 2013). Therefore, it is important to validate the DTQ in adolescents because of the development of metacognition during this phase and before the maturity of the capacity to reflect on one's own thoughts and behaviours.

Although the ICD-11 includes CSBD (classification number 6C72) as an impulse control disorder (Kraus et al., 2018) several scholars have conceptualised it as an addictive behaviour (Kraus, Voon, & Potenza, 2016; Potenza, Gola, Voon, Kor, & Kraus, 2017). Addictions typically comprised activities conducted repeatedly, habitually, and compulsively and interfere in major areas of life functioning (Miller, Forcehimes, & Zweben, 2011). They may involve chronic relapsing, feelings of tension or arousal before committing the act, and subsequent pleasure, gratification, or relief at the time of committing the act. Behaviours often become less pleasurable and more habitual over time. They may be driven by negative reinforcement and involve craving states (Grant, Potenza, Weinstein, & Gorelick, 2010).

CSBD parallels these tendencies in various ways: individuals with CSBD often develop anxiety and depression symptoms when not engaging in sexual behaviour (e.g. Wines, 1997); They also report difficulties in attempting to stop or reduce the frequency of sexual activities, and spending excessive amounts of time engaging in sexual activities (Bőthe et al., 2020). Two key cognitive and behavioural components that relate to CSBD and other addictive behaviours are thought suppression (e.g. Erskine et al., 2012; Klein, 2007; Riley, 2014) and impulsivity (Hartmann, Czaja, Rief, & Hilbert, 2010; Hu, Zhen, Yu, Zhang, & Zhang, 2017; Rømer Thomsen et al., 2018; Wetterneck, Burgess, Short, Smith, & Cervantes, 2012).

### Thought suppression and impulsivity in addictive behaviours

Thought suppression is a mental control strategy, whereby one attempts to manage emotional distress by attempting to keep certain unwanted thoughts out of awareness (Wenzlaff & Wegner, 2000). For example, individuals who present with CSBD report difficulties dealing with repetitive sexual thoughts. These thoughts make them feel that “something might be wrong” with them, and they believe that such thoughts may harm their daily functioning and even increase their need for sex as a means of relaxation. One common strategy to handle unwanted thoughts is to try to suppress them (e.g., Brockman, Ciarrochi, Parker, & Kashdan, 2017), particularly if a person cannot openly share these thoughts with others (Gross & John, 2003). The engagement in thought suppression, however, can paradoxically lead to an increase in the suppressed thought (Abramowitz, Tolin, & Street, 2001; Wenzlaff & Luxton, 2003). This effect has been observed in CSBD (Efrati, 2019), alcohol use (Klein, 2007), cigarette use (Erskine et al., 2012) and problem gambling (Riley, 2014), which suggests that there is an underlying relationship with addictive behaviours (Spada, Caselli, Nikčević, & Wells, 2015).

Impulsivity refers to a series of behaviours that are poorly planned and prematurely executed with little forethought to the consequences and is closely linked, but distinct from compulsivity, which involves more conscious deliberation (Dalley, Everitt, & Robbins, 2011). Similar to thought suppression, impulsivity has also been associated with various problematic behaviours, including alcohol consumption (Castellani & Rugle, 1995), loss-of-control eating (Hartmann et al, 2010), online gaming (Hu et al., 2017), Internet pornography use (Wetterneck et al., 2012) and problematic sexual behaviours (Bőthe et al., 2019). A recent systematic meta-review concluded that impulsivity may be considered a core process that underpins both substance and behavioural addictions in varying degrees (Lee, Hoppenbrouwers, & Franken, 2019). The link between impulsivity and CSBD is not conclusive (Efrati & Gola, 2019a). Several studies have found links between CSBD and self-report and/or task-related measures of impulsiveness (Antons & Brand, 2018; Miner, Raymond, Mueller, Lloyd, & Lim, 2009; Reid, Garos, & Carpenter, 2011; Voon et al., 2014; Walton, Cantor, Bhullar, & Lykins, 2017; Zilberman, Yadid, Efrati, Neumark, & Rassovsky, 2018). Conversely, Walton et al., (2017) found that only one-third of individuals with CSBD have impulsivity scores above the range of normal impulsivity.

### Aims of our study

The current research had three aims. The first aim was to validate a Hebrew version of the DTQ (Caselli & Spada, 2011), specifically among adolescents – and understudied population which preliminary reports show a high percentage of CSBD as compared to adults (although these age groups have never been contrasted to date). The second aim was to determine the predictive capacity of the DTQ for CSB independently of the established related cognitive and behavioural dispositions – thought suppression and impulsivity. To ascertain whether the DTQ would be associated with the addictive nature of CSB and not with its negative affectivity or with the general association with psychopathology, we also controlled for depression, anxiety, and stress. The third aim was to explore the association between desire thinking and CSB that was found among adults in adolescents.

Study 1: Translation and adaptation of a Hebrew Version of the Desire Thinking Questionnaire (DTQ-H)

## Method

### Participants

A convenience sample of 718 adolescents (464 female; mean age = 15.83 years [SD = 1.36; range 14–18 years]) was recruited for this study and completed the preliminary version of the Hebrew version of the DTQ-H. 93.4% of the sample were born in Israel, with 65% classifying themselves as secular and 35% as religious.

### Materials

The items from the original DTQ (Caselli & Spada, 2011) were translated into Hebrew by a speaker proficient in both languages and then back translated by the first author. The original questionnaire contains 10 items and has a two-factor structure (Verbal Perseveration & Imaginal Prefiguration). Items were framed as statements to which participants could respond to on a four-point Likert-type scale to indicate their level of agreement (“1. Almost never”, “2. Sometimes”, “3. Often”, and “4. Almost Always”). The items were preceded by a pre-amble in Hebrew that read as follows:

“Youth feel a strong desire to do something different in their minds. Please read the statements and circle the number that describes the experience you experience when you feel a strong desire to perform some activity. In your answer, please refer to how you act in reality and not in the manner that you tried to drive it. There is no right or wrong answer.”

### Procedure

The study comprised of a convenience sample, recruited by postings on bulletin boards and online forums for volunteers for research on sexuality among 14- to 18-year-old adolescents. The inclusion criterion was based solely on age. No exclusion criteria were used. The questionnaires were uploaded to Qualtrics (Qualtrics, 2019). After adolescents responded and agreed to participate, their parents were contacted (by e-mail and/or phone) and were asked to review the questionnaires (87% agreed to participate). If the parents approved, they were asked to sign an informed parental consent form and e-mail it to a research assistant. Following parental consent, a link for the online survey was sent to the adolescent, who was assured of the anonymity of the survey. Participants were then asked to complete the survey at home, without anyone else present. Each adolescent was asked to sign an informed consent form prior to beginning work on the questionnaire. Once the questionnaire had been submitted, the researchers followed up with the adolescents with an online debriefing. Finally, the participants were thanked for being part of the research. Overall, completion time was 10 min, on average.

### Analysis

The ten original items of the DTQ were subjected to an EFA with principle axis factoring using SPSS (version 25; IBM Corp, 2017). A promax rotation with Kappa set to 4 was chosen. It was decided a priori that items that loaded less than 0.4 on either factor would be discarded, as would be items that loaded above 0.4 on both factors. If, however, an item loaded more than 0.4 on only one factor, but the second factor loading was within 0.2 of the loading on the first factor, it would also be discarded. For example, if a factor loaded 0.5 on the first factor, it would be discarded if the loading on the second factor was above 0.3. This protocol was used in order to exclude items that influenced both factors and mirror that used in the original DTQ validation study (Caselli & Spada).

### Ethics

The study was approved by Beit-Berl College Center's Institutional Review Board. Informed consent forms and parental consent were signed before the onset of the study.

## Results

### Exploratory Factor Analysis

Similar to the results of the original DTQ, the EFA led to a two-factor solution (eigenvalues of 5.29 and 1.17), which accounted for 56.26% of the variance and the estimated correlation between the two factors was 0.68 (Table 1 shows the factor loadings of the individual items). One of the items (#8), loaded on both factors and was thus discarded. Reliability indices were determined by computing Cronbach's alpha for the remaining 9 items. This coefficient was 0.90 for the total score, 0.85 for Factor 1 and 0.84 for Factor 2.

Study 2: Validation of the DTQ-H

**Table 1.** Factor loadings from exploratory factor analysis

		Factor 1	Factor 2
1. I imagine myself doing the desired activity	אני מדמיין את עצמי עושה את הפעילות הנחשקת	−0.18	0.94
2. I imagine how I would feel like when engaging in the desired activity	אני מדמיין איך אני ארגיש כאשר אשתתף בפעילות הנחשקת0	0.03	0.79
3. I anticipate the sensations I would feel practicing the desired activity	אני מנסה לחזות את התחושות שארגיש כשאשתתף בפעילות הנחשקת	0.08	0.75
4. If I were not practice the desired activity for a long time, I would think about it continuously	אם לא אבצע את הפעילות הנחשקת זמן רב, אחשוב עליה ללא הפסקה	0.60	0.14
5. When I begin to think about the desired activity I find it difficult to stop	כאשר אני מתחיל לחשוב על הפעילות הנחשקת, קשה לי להפסיק	0.67	0.09
6. When I begin to think about the desired activity I continue until I manage to engage in it	כאשר אני מתחיל לחשוב על פעילות נחשקת אני ממשיך לחשוב עליה עד שאני מצליח לבצע אותה	0.81	−0.10
7. I repeat mentally to myself that I need to practice the desired activity	אני כל הזמן מזכיר לעצמי בראש שעלי לעשות את הפעילות הנחשקת	0.72	0.01
8. I begin to imagine the desired activity every time it comes to my mind	אני מתחיל לדמיין את הפעילות הנחשקת כל פעם שהיא עולה לי בראש	0.40	0.43
9. I imagine myself involved in the desired activity as if it were a movie	אני רואה את עצמי לוקח חלק בפעילות הנחשקת, כאילו כחלק מסרט	0.24	0.47
10. My mind is focused on repeating what I desire till I manage to satisfy it	הראש שלי ממוקד כל הזמן בפעילות הנחשקת עד שאני מצליח לספק את הרצון	0.75	–0.06

## Introduction

In order to validate the DTQ-H we: (1) determined construct validity (by running a Confirmatory Factor Analysis; CFA); (2) established concurrent validity (by observing whether the two factors of the DTQ-H would correlate significantly with established measures of impulsivity, thought suppression and negative affect); (3) examined internal reliability; (4) examined incremental validity by observing whether the DTQ-H would predict levels of behaviour when controlling for negative affect, thought suppression and impulsivity.

## Method

### Participants

A convenience sample of 379 adolescents (217 female; mean age = 16.27 years [SD = 1.17; range 14–18 years]) completed a battery of online questionnaires. Eligibility matched that employed in Study 1. Of this sample, 95.5% were born in Israel and 59.4% considered themselves to be secular, while 40.6% considered themselves religious.

### Materials

#### Impulsiveness measure

The Barratt Impulsiveness Scale-11 (BIS-11; Patton, Stanford, & Barratt, 1995; translated to Hebrew by Glicksohn, Leshem & Aharoni, 2006) is a 30-item measure of impulsivity using a 4-point Likert scale (1 – *Rarely/Never*, 4 – *Almost always/Always*). It comprises three subscales: attentional impulsiveness (e.g., “I get easily bored when solving thought problems”), motor impulsiveness (e.g., “I do things without thinking”) and non-planning impulsiveness (e.g., “I am more interested in the present than the future”). However, there is evidence to suggest that these three subscales are not clearly defined across cultures (Vasconcelos, Malloy-Diniz, & Correa, 2012). Patton and colleagues (1995) report internal consistency coefficients for the BIS-11 total score that range from 0.79 to 0.83 for various clinical and non-clinical populations. Cronbach's alpha of the total score in this sample was 0.79.

#### Thought suppression measure

A version of the Food Thought Suppression Inventory (FTSI; Barnes, Fisak, & Tantleff-Dunn, 2009) was adapted for this study by back-to-back translation procedure (from English to Hebrew and back). For each of the references to thoughts about food, the authors substituted these with thoughts about sex. The FTSI is a 15-item, unidimensional measure of the tendency to avoid food-related thoughts (e.g., “There are images about food that come to mind that I cannot erase”). (1 – *Strongly disagree*, 5 – *strongly agree*). Cronbach's alpha was reported as 0.96 in a population of women (Barnes et al., 2009) and 0.95 in a population of men (Barnes & White, 2010). Cronbach's alpha in this current sample was 0.93.

#### Behaviour measure

The Individual-based Behaviour Scale (I-CSB; Efrati & Mikulincer, 2018; originally developed in Hebrew) was developed to assess distinct aspects of CSB, such as sexual fantasies, obsessive sexual thoughts, and spending a great deal of time watching pornography. The I-CSB is a self-report questionnaire with 24 items measuring the following factors: Unwanted consequences (e.g., “I feel that my sexual fantasies hurt those around me”); lack of control (e.g., “I waste lots of time with my sexual fantasies”); negative affect (e.g., “I feel bad when I don't manage to control my sexual urges”); and affect regulation (e.g., “I turn to sexual fantasies as a way to cope with my problems”). Using a 7-point Likert scale, participants were asked to rate the degree to which each statement is descriptive of their feelings (1 – *not at all*, 7 – *very much*). The questionnaire was successfully used in previous research on non-clinical populations of adults and adolescents (Efrati & Gola, 2019b), and on clinical populations of Sexaholics Anonymous Twelve-Step program patients (Efrati, Gerber, & Tolmacz, 2019; Efrati & Gola, 2018; Efrati & Mikulincer, 2018). Cronbach's alphas were 0.93 for unwanted consequences, 0.94 for lack of control, 0.88 for negative affect, and 0.91 for affect regulation. We also computed a total I-CSB score by averaging the 24 I-CSB items (Cronbach's alpha = 0.97). The I-CSB measure has a clinical cut-off of 4.1 in the I-CSB total score (Efrati & Mikulincer, 2018). In this sample Cronbach's alpha was 0.94.

#### Negative affect measure

The short form of the Depression Anxiety Stress Scale (DASS-21; Antony, Bieling, Cox, Enns, & Swinson, 1998; translated to Hebrew by Doron, Derby, Szepsenwol, & Talmor, 2012a,b) is a 21-item measure using a 4-point Likert scale that assesses general symptoms of psychopathology. The DASS-21 distinguishes between depression, physiological arousal and psychological agitation. It has acceptable reliability and has been validated using clinical and non-clinical populations. It contains three orthogonal factors (depression (DASS-D), anxiety (DASS-A) and stress (DASS-S) as well as an overall factor of psychological distress (DASS-T) (Henry & Crawford, 2005). Cronbach's alpha of the DASS-T in this sample was 0.95.

### Procedure

This followed the same structure as in Study 1. In Study 2, 76% of the parents signed parental consent. The inclusion criterion was based solely on age (14–18 years). No exclusion criteria were used. The order of the questionnaires was as follows: DTQ, I-CSB, BIS-11, Sexual Thought Suppression Inventory, DASS-21 and sociodemographic measures (age, gender, religiosity). The study was approved by Beit-Berl's Institutional Review Board (IRB) committee. Overall, completion time was 25 min, on average.

### Analysis

A CFA was performed on the data obtained from the participants using jamovi, which is built on lavaan in R (R Core Team, 2013; Rosseel, 2012; The jamovi project, 2019). We defined the latent variables as verbal perseveration and imaginal prefiguration and assumed a covariance between them. The remaining 9 items were considered as congeneric indicators of the latent variables. We utilised five indices to evaluate the fit of the model: a Chi-square measure of fit, the Comparative Fit Index (CFI), the Tucker-Lewis Index (TLI: also known as the Non-Normed Fit Index), the Standarized Root Mean Square Residual (SRMR) and the Root Mean Square Error of Approximation (RMSEA). Following the suggestions set out by Byrne (2001) and Hu and Bentler (1999), the cutoffs utilised in this study were a ***χ***^2^**/**d*f* of less than 2, 0.95 for the CFI and TLI, 0.08 for the SRMR and 0.06 for the RMSEA.

Reliability analysis was conducted by calculating Cronbach's alpha Concurrent validity were ascertained by calculating correlations between the subscales of the DTQ-H and various measures and and incremental validity utilised a hierarchical regression to predict levels of behaviour when controlling for various measures. All of these analyses were done using SPSS (version 25; IBM Corp, 2017).

## Results

### Confirmatory Factor Analysis

The initial 9-item CFA assumed a covariance between the latent variables and resulted in a mixed fit: the chi-square test was significant (***χ***^2^ = 123, df = 26, *P* > 0.001) and the ***χ***^2^**/**df = 4.73. This model generated a CFI of 0.95, a TLI of 0.93, a SRMR of 0.072 and an RMSEA of 0.0995 (*P* < 0.001). Parameter estimates were reviewed, and modification indices were calculated (see Table 2). Together these suggested a re-specified model, where the top 3 cross-correlating items were removed (#5, 6, 9). The re-specified model retained the covariances between latent variables and acceptable fit of the data was demonstrated. Although the Chi-square test remained significant (***χ***^2^ = 16, df = 8, *P* < 0.05), the resulting ***χ***^2^**/**df = 2 suggests acceptable fit (Byrne, 2001). This new model also yielded the following results: CFI of 0.99, TLI of 0.99, SRMR of 0.029 and RMSEA of 0.051 (*P* > 0.05), demonstrating acceptable construct validity. Based on these results, the DTQ-H was confirmed as having two correlated factors, verbal perseveration (DTQ-H-VP; 3 items) and imaginal perseveration (DTQ-H-IP; 3 items).

**Table 2.** Modification Indices for the DTQ-H

	DTQ-H-IP	DTQ-H-VP
Question 1		1.191
Question 2		13.034
Question 3		0.736
Question 9		48.182
Question 4	7.256	
Question 5	24.910	
Question 6	14.510	
Question 7	3.078	
Question 10	0.513	

*Note*: DTQ-H-VP = Hebrew Version of the Desire Thinking Questionnaire-Verbal Perseveration; DTQ-H-IP = Hebrew Version of the Desire Thinking Questionnaire-Imaginal Prefiguration.

### DTQ-H subscale reliability

Cronbach's alpha (Cronbach, 1951) was calculated using jamovi, which utilises the psych package for R (R Core Team, 2013; Revelle, 2019; The jamovi project, 2019). The DTQ-H-VP (verbal perseveration) consisted of 3 items (*α* = 0.79), demonstrating acceptable levels of reliability. The DTQ-H-IP demonstrated good levels of reliability (3 items; *α* = 0.88).

### Concurrent validity

Table 3 shows the means, standard deviations, ranges and bivariate correlations for all the study variables. A series of Shaprio–Wilk tests of normality were conducted on the data, which suggested that all measurements were significantly different than normal, aside from the Barrett Impulsivity Scale. As a result, a series of non-parametric, Spearman's Rho correlation analyses were conducted on the data (see Table 3). These revealed that the DTQ-H-VP and DTQ-H-IP were moderately correlated with each other (rs = 0.47, *P* < 0.01). There were, however, no demographic relationships, as age, gender and religious affiliation did not correlate with either subscale.

**Table 3.** Means, standard deviations, skewness, kurtosis and bivariate correlations

	Mean	S.D.	Skewness	Kurtosis	1	2	3	4	5	6	7	8
1. Gender	–	–										
2. Religion	–	–			−0.02							
3. Age	16.27	1.17	−0.17	−0.81	0.01	−0.01						
4. DTQ-H-VP	6.12	2.63	0.51	−0.78	−0.04	0.01	0.01					
5. DTQ-H-IP	8.25	2.72	−0.17	−0.97	−0.07	0.01	0.05	0.47^**^				
6. I-CSB	61.86	29.49	0.60	−0.30	−0.27^**^	0.21^**^	−0.03	0.37^**^	0.25^**^			
7. TSI	30.36	12.05	0.41	−0.72	−0.22^**^	0.34^**^	−0.05	0.28^**^	0.27^**^	0.69^**^		
8. BIS	65.99	10.77	0.13	0.01	0.04	−0.10	−0.06	0.21^**^	0.02	0.26^**^	0.14^*^	
9. DASS-T	60.05	15.88	−0.33	−0.71	−0.21^**^	0.03	0.06	−0.10^*^	−0.08	−0.23^**^	−0.22^**^	−0.39^**^

*n* = 345; ^*^P < 0.05; ^**^P < 0.01.

*Note*: Gender = Gender of participants (0 = “male”; 1 = “female”); Religion = Religious Affiliation (0 = “secular”; 1 = “religious”); Age = Age in years; DTQ-H-VP = Hebrew Version of the Desire Thinking Questionnaire-Verbal Perseveration; DTQ-H-IP = Hebrew Version of the Desire Thinking Questionnaire-Imaginal Prefiguration; I-CSB = Individual-based Compulsive Sexual Behaviour Scale; TSI = Thought Suppression Inventory; BIS = Barratt Impulsiveness Scale; DASS-T = Depression, Anxiety Stress Scale-21 (Total); *n* = 345.

The DTQ-H-VP was significantly correlated with each of the other measures. There was a weak positive correlation with impulsivity (rs = 0.21, *P* < 0.01) and thought suppression (rs = 0.28, *P* < 0.01) and a weak negative correlation with negative affect (rs = −0.10, *P* < 0.05). Lastly, there was a moderate positive correlation with behaviours (rs = 0.37, *P* < 0.01). The DTQ-H-IP demonstrated a weak positive correlation with thought suppression (rs = 0.27, *P* < 0.01) and behaviour (rs = 0.25, *P* < 0.01).

### Incremental validity

Incremental validity was ascertained by performing a regression analysis in which the I-CSB was the dependent variable and the predictor variables were entered in the following order: gender and religious affiliation, which were correlated with the I-CSB (see Table 3), negative affect, impulsivity and thought suppression, and then the two factors of the DTQ-H. Assumptions were checked by reviewing the distribution of the residuals and ensuring homoscedasticity. Both were deemed to be acceptable. Following the first three steps, the demographics, DASS, BIS and TSI accounted for a significant amount of the variance (*R*^2^ = 0.51, *P* < 0.001). The addition of the DTQ-H resulted in a significant change (*R*^2^ = 0.54, *P* < 0.001). However, the DTQ-H-VP subscale was a predictor of levels of individual-based behaviour after controlling for the other factors (Beta = 0.193, *P* < 0.001), but the DTQ-H-IP was not. In this model, religious affiliation and negative affect were non-significant predictors of behaviour (see Table 4).

**Table 4.** Regression coefficients for the DTQ-H, accounting for gender, religious affiliation, affect, impulsivity and thought suppression. Criterion variable: I-CSB

Model		Coefficients^a^						
		Unstandardised coefficients		Standardised coefficients	*t*	Sig.	95.0% Confidence interval for B	
		B	Std. error	Beta			Lower bound	Upper bound
1	(Constant)	73.211	6.786		10.788	<0.001	59.863	86.559
	Gender	−16.183	3.054	−0.271	−5.299	<0.001	−22.189	−10.177
	Religion	10.086	3.066	0.168	3.290	0.001	4.055	16.117
2	(Constant)	112.284	9.101		12.337	<0.001	94.382	130.186
	Gender	−19.794	2.964	−0.332	−6.678	<0.001	−25.625	−13.964
	Religion	10.331	2.916	0.172	3.542	<0.001	4.595	16.068
	DASS-T	−0.561	0.092	−0.302	−6.089	<0.001	−0.743	−0.380
3	(Constant)	13.937	12.561		1.110	0.268	−10.771	38.645
	Gender	−9.138	2.437	−0.153	−3.750	<0.001	−13.931	−4.345
	Religion	−1.735	2.482	−0.029	−0.699	0.485	−6.617	3.148
	DASS-T	−0.146	0.080	−0.079	−1.185	0.070	−0.304	0.012
	BIS	0.426	0.114	0.156	3.751	<0.001	0.203	0.650
	TSI	1.496	0.108	0.611	13.854	<0.001	1.284	1.708
4	(Constant)	7.782	12.414		0.627	0.531	−16.638	32.201
	Gender	−9.423	2.367	−0.158	−3.980	<0.001	−14.080	−4.767
	Religion	−0.374	2.418	−0.006	−0.155	0.877	−5.131	4.382
	DASS-T	−0.152	0.078	−0.082	−1.954	0.052	−0.304	0.001
	BIS	0.350	0.111	0.128	3.138	0.002	0.131	0.569
	TSI	1.349	0.110	0.551	12.216	<0.001	1.132	1.566
	DTQ-H-VP	2.162	0.479	0.193	4.512	<0.001	1.219	3.104
	DTQ-H-IP	0.163	0.462	0.015	0.352	0.725	−0.747	1.072

*Note*: Gender = Gender of participants (0 = “male”; 1 = “female”); Religion = Religious Affiliation (0 = “secular”; 1 = “religious”); DASS-T = Depression, Anxiety Stress Scale-21 (Total); TSI = Thought Suppression Inventory; BIS = Barratt Impulsiveness Scale; DTQ-H-VP = Hebrew Version of the Desire Thinking Questionnaire-Verbal Perseveration; DTQ-H-IP = Hebrew Version of the Desire Thinking Questionnaire-Imaginal Prefiguration; I-CSB = Individual-based Compulsive Sexual Behaviour Scale; *n* = 345.

a Dependent Variable: I-CSB.

## Discussion

The aim of our two studies was to investigate psychometric properties of the Hebrew version of the DTQ and explore its potential association with behaviour in adolescent independently of negative affect, thought suppression and impulsivity. The results of our studies confirmed the two-factor solution of DTQ-H, allowing for the distinction between imaginal prefiguration and verbal perseveration. The scale also evidenced a good internal consistency, reliability and concurrent validity in line with Caselli's and Spada's (2011) original version of DTQ. It also extends the concurrent validity of the measure by comparing it with other two core constructs that are involved in addictive behaviours: thought suppression and impulsivity.

In the second study we also assumed that desire thinking may be associated to CSB in adolescents. Our findings showed that, controlling for negative affect, impulsivity and thought suppression, verbal perseveration predicted levels of CSB. These findings align themselves with those observed across several addictive behaviours (Caselli & Spada, 2015; Mansueto et al., 2019) and support views in the field that CSB may arise from a combination of dysfunctional cognitive regulation strategies to cope with desire-related intrusions (e.g. verbal perseveration or thought suppression) and impulsivity traits. The specific finding that imaginal prefiguration was not a significant predictor of CSB aligns itself with research indicating that this factor may be more proximal to craving (Caselli, Soliani, & Spada, 2013; Martino et al., 2017, 2019) and that verbal perseveration may mediate its relationship with engagement in addictive beahviour as highlighted by Allen et al. (2017) who using mediation analysis found that the effect of imaginal prefiguration on pornography craving was mediated by verbal perseveration.

The question that arises is why would desire thinking be an independent predictor of CSB? A possible explanation is that the repetitive self-talk regarding the need satisfy desires (the verbal perseveration component of desire thinking) can be considered as a form of sustained mis-regulation strategic coping. The function of desire thinking may thus be that of helping to regulate, in the short-term, discrepancies between actual and ideal states with resultant anticipation of pleasant states and relief from emotional distress (Caselli & Spada, 2010, 2016; Kavanagh, Andrade, & May, 2004, 2005). In the medium to longer term, however, the activation of desire thinking will inevitably lead to an escalation and perseveration of sexual arousal as the desired target (in this case sexual activity) is perseveratively elaborated upon but not achieved. This, in turn, could lead to the desired activity being perceived as the only, and increasingly urgent, route to regulate sexual arousal and associated distress. A second route to addictive behaviours may involve the role of verbal perseveration in solidifying on-line rate of conviction in permissive beliefs that may act as motivational drive for decision to approach the desire target (Caselli & Spada, 2016).

The current study is the first, to the best of our knowledge, that has linked desire thinking with CSB among adolescents. Among adults, imaginal prefiguration (allocation of attentional resources and mental imagery elaboration regarding sexual content and behaviour) and verbal perseveration (extended self-talk regarding meaningful reasons for engaging in sexual-related activities) were strongly linked with pornography craving. We have found, however, that among adolescents, though both imaginal prefiguration and verbal perseveration were linked with CSB only verbal perseveration predicted the severity of CSB when controlling for all other variables. A possible explanation for this is that pornography craving does not equate to CSB, which is comprised not only craving but multiple behaviours and sensations. All of which might be preceded by sexual-related mental imagery (e.g. fantasies) but not always by self-talk regarding meaningful reasons for engaging in these behaviours. For instance, one facet of CSB is lack of behavioural control that refers to constant uncontrolled engagement with sexual fantasies, urges, and behaviours with numerous unsuccessful efforts to significantly reduce repetitive sexual behaviour. In other words, people with CSB try to control and/or reduce the behaviour and fail to do so. This may be linked to the presence of verbal perseveration. Future research might explore these options in greater depth.

There are numerous clinical and health implications that arise from these findings. For example, it may be helpful to include psychoeducational activities that help adolescents to identify desire thinking as a form of coping that worsens negative affect, hinders the pursue of long-term valued goals and exacerbates degree of risky behaviours (Caselli & Spada, 2013; Spada, Caselli, & Wells, 2013). In terms of clinical interventions, Metacognitive Therapy (Wells, 2000) may provide valuable techniques aimed at interrupting desire thinking, including attention training, detached mindfulness and postponement exercises. Re-appraising the beliefs about the benefits and uncontrollability of desire thinking may also be of value (Caselli & Spada, 2016).

This study suffers from the typical limitations of cross-sectional designs based on self-report data such as possible errors in measurement and the preclusion of causal inferences. Future studies might manipulate the level of desire thinking (imaginal prefiguration and/or verbal perseveration) such as by ways of guided imagery and examine whether state level of sexual fantasies and action tendencies change in response to the different conditions. Furthermore, the presence of concurrent psychological disorder was not assessed, however controlling for anxiety and depression should provide a degree of confidence in the specificity of the results.

Directions for future research include investigating possible causal relationships between desire thinking and behaviour and its longitudinal impact in facilitating risky behaviours. Explicating the nature of the observed relationships using ecological momentary assessment and experimental designs, which will disentangle antecedents from consequences and provide information regarding the accuracy of desire thinking in predicting decision to approach, would also be needed.

### Concluding remarks

In conclusion, the DTQ-H appears to be a reliable and valid measure of desire thinking in adolescents and appears to predict CSB independently of established constructs. It may serve as a viable measure to explore in more depth the development and maintenance of sexual-related meta-cognitions that were found to be highly predictive of sexual addiction and various other addictive behaviours among adults.

## Funding sources

None.

## Authors' contribution

YE conducted the study and edited the first draft of the study. DCK analysed the results and wrote the first draft of the study. MMS and GC edited the paper and suggested critical theoretical and empirical additions to the paper.

## Conflict of interest

Authors have no conflict of interest.
